# The Fragmented Nature of Biosensor Development: Challenges and Paths to Mitigation

**DOI:** 10.3390/bios16060341

**Published:** 2026-06-16

**Authors:** Gil Zimran, Assaf Mosquna

**Affiliations:** The Robert H. Smith Institute of Plant Sciences and Genetics in Agriculture, The Hebrew University of Jerusalem, Rehovot 7610000, Israel

**Keywords:** biosensor development, disciplinary fragmentation, developmental record, Library re-use, BLR-tables

## Abstract

Genetically encoded biosensors are now central tools, deployed either as intracellular reporters to advance basic research, or as whole-cell reagents that detect analytes in diverse sample-types. Across the diversity of molecular scaffolds and modes of operation, biosensors serve a common functional purpose: translating ligand presence into a readable signal. Despite this shared logic, biosensor development as a field of practice remains fragmented: different scaffolds and modalities are advanced in separate, often lab-specific pipelines with diverse assays, metrics, and design practices. Moreover, libraries, selection histories and performance data generated during routine campaigns rarely outlive the projects that produced them. In this perspective, we focus on this fragmentation as a field-level bottleneck and argue that it deserves explicit attention in its own right. We discuss how modest, incremental steps—such as structured development records, adherence to high-information screening formats, library annotation, and community-level deposition infrastructure—could make biosensor development more reproducible, more comparable, and easier to build on across projects and laboratories. We further argue that such infrastructure will become increasingly valuable as computational protein design matures—not as a competing approach, but as the source of diverse, comparable, and context-annotated experimental data that sequence-function models and design benchmarks ultimately depend on.

## 1. Introduction

Over the past two decades, genetically encoded biosensors have been developed to report a wide range of biochemical targets and are now common tools in many areas of biology, biotechnology, and medicine. These sensors support a broad spectrum of uses by coupling ligand recognition to a measurable signal in living cells. In applied and analytical settings, whole cells harboring a genetically encoded sensor (i.e., whole-cell biosensors) are mixed directly with a sample, and the resulting signal—typically fluorescence or a growth response—reports analyte concentration in a format that requires no purified protein and can in principle be deployed outside the laboratory [1,2]. In basic research, genetically encoded biosensors report the dynamics of metabolic and signaling pathways, biochemical states, or ion fluxes with spatial and temporal resolution that bulk measurements cannot provide [3,4,5]. In a bioengineering context, they function as internal measurement and selection devices, linking intracellular biochemical performance to growth or fluorescence outputs to guide the optimization of proteins, metabolic pathways and genetic circuits [6]. Across these different settings, their shared role is to convert otherwise hidden biochemical states into quantitative signals that can be measured and, in many cases, used to steer experimental, clinical, and regulatory decisions.

At the mechanistic level, this shared role is realized by a diverse family of architectures that differ in how analytes are detected and how signals are relayed to an output. Transcription factor–based biosensors couple ligand binding to reporter gene expression through changes in DNA binding, transcriptional regulation, or nuclear localization [7,8]; these, along with riboswitch-based sensors, are particularly prevalent in whole-cell biosensor applications, where compact, genetically encoded circuits must function reliably and consistently in microbial hosts. Riboswitch-based sensors carry analogous function at the RNA level, relying on structural elements whose ligand-induced conformational changes affect translation initiation, splicing, or RNA stability [9,10,11]. Ligand-gated receptors, including GPCR-based sensors, couple binding to downstream signaling or reporter activation [5,12]. Split-protein systems use ligand-dependent protein–protein interactions to reconstitute functional complexes—from inactive fragments—to activate reporter activity in a dose-responsive manner. These include, for example, transcription factors, CRISPR-Cas systems or proteases that regulate downstream reporters, and luciferases that provide a direct bioluminescent readout [13,14,15,16,17]. Direct optical readout is also a defining feature of fluorescent protein–based biosensors, which dispense with enzymatic substrates entirely: ligand binding can reconstitute a functional fluorescent protein in split-FP designs [18,19], or modulate fluorescent properties in FRET- or single-FP sensors by changing fluorophore distance, orientation, or local environment [20,21,22].

**Figure 1:**
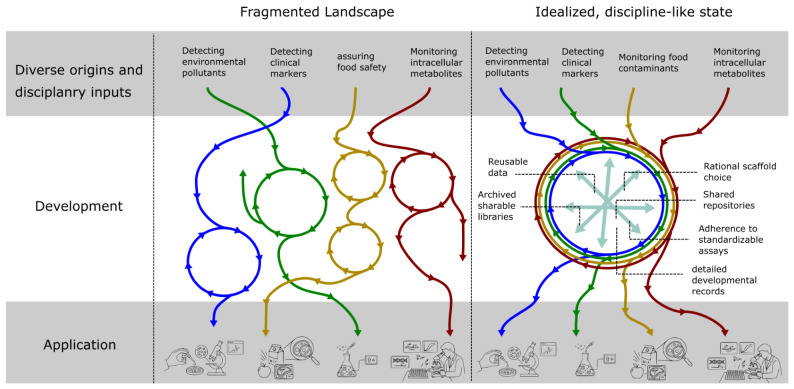
Schematic contrast between the current fragmented landscape of genetically encoded biosensor development and an idealized, more discipline-like state. Different colors represent distinct development lineages—reflecting the disciplinary diversity and scaffold-specific traditions described in the text. In the idealized state (right), this diversity converges through a shared developmental infrastructure before diverging again into applications. The shared hub does not impose uniformity on the science; rather, it provides the connective tissue that allows independent campaigns to inform and build on one another, gradually making the field more cumulative.

Despite this diversity of architectures, hosts, and applications, the primary task that most genetically encoded biosensors are designed to perform is conceptually straightforward: to establish a quantitative, reasonably monotonic relationship between the level or activity of a target analyte and a readable signal. In principle, this simple functional goal should lend itself to shared ways of designing, characterizing, and reporting sensor performance, so that different scaffolds and hosts could plug into a common measurement logic. In practice, however, the diversity of mechanisms is mirrored by an equally diverse set of design choices, screening assays, and reporting conventions, so that many biosensors are developed in isolation within lab-specific pipelines that are difficult to compare, generalize, or reuse (Figure 1).

In this perspective, we use the term “fragmented biosensor development” to refer to this scattered landscape of workflows and standards, and we argue that it constitutes a central bottleneck for the field that deserves explicit attention in its own right—one with particular consequences for whole-cell biosensor development, where the potential to systematically expand analyte coverage and reuse validated microbial chassis remains largely unrealized. The following sections describe how this fragmentation arises from disciplinary dispersion, modality-specific traditions, and project-level pipeline choices, and discuss how modest moves toward shared infrastructure and reporting could bring biosensor development closer to the underlying simplicity of its measurement task.

## 2. Why Biosensor Development Resists Convergence

### 2.1. Disciplinary Dispersion: Purpose-Driven and Opportunistic Origins

Genetically encoded biosensors are developed and used across many different areas of research rather than within a single, tightly defined discipline, and this dispersion is one of the upstream reasons why development remains fragmented. Metabolic engineers build sensors to monitor and optimize production strains [6,23,24], plant biologists engineer sensors for hormones or metabolites *in planta* [3,25], microbiologists repurpose regulatory circuits from pathogens and environmental organisms to report on infection-relevant or ecologically important signals [26], and neurobiologists design reporters for neurotransmitters and neuromodulators [27]. Naturally, this diversity is reflected in how biosensor development campaigns are designed and communicated. However, while much of this divergence is constructive—as different downstream applications entail different constraints and prioritize different performance parameters—the field of biosensor development stands to benefit from a greater degree of crosstalk centered around their shared core functionality.

In many of these settings, biosensors arise opportunistically: a lab studying a particular regulatory protein, RNA element, or signaling pathway realizes that the same component could be reconfigured as a sensor, or that a simple reporter would remove a bottleneck in an existing assay. In other cases, biosensor projects are more purpose-driven—for example, when metabolic engineering or synthetic biology groups set out explicitly to construct sensors for pathway optimization or dynamic control, when whole-cell biosensor efforts target specific environmental or food-safety analytes for field deployment, or when clinical and diagnostic efforts target specific analytes for translation. But even these more intentional undertakings are usually scoped around a particular host, target, and question, and once a working sensor has been demonstrated, attention tends to return to the underlying biological or engineering goal. For instance, when a microbial whole-cell biosensor developed for a specific contaminant is published, the scaffold and selection history it relied on are rarely preserved in a form that would let others retarget it toward alternative analytes. As a result, building on and generalizing beyond a given project remains challenging, and many genetically encoded biosensors persist as one-off constructions tied to specific labs and problems rather than as contributions to a shared, cumulative engineering discipline. This project-level isolation underlies many of the downstream issues discussed in the following sections.

### 2.2. Diverse Configurations and Modalities

Genetically encoded biosensors do not form a single, unified technological family; instead, several main configurations have largely developed along their own trajectories, with distinct hosts, targets, benchmarks, and even vocabulary. Transcription factor–based biosensors, for example, are most deeply entrenched in microbial synthetic biology and metabolic engineering, where bacterial regulators wired to promoters provide quantitative readouts of intracellular metabolite levels or pathway intermediates—and in some cases feed these signals directly back into metabolic circuits to enable autonomous, dynamic pathway control [8,23,28]. In this community, core design questions are often framed in terms of dynamic range, response threshold, leakiness, and control of transcriptional and translational tuning elements such as promoters and ribosome-binding sites, typically in hosts like *Escherichia coli* or *Saccharomyces cerevisiae* [29]. By contrast, riboswitch-based biosensors are discussed in terms of RNA folding kinetics, aptamer–effector specificity, and regulatory architectures that couple ligand binding to translation or splicing, with much of the jargon and design intuition drawn from RNA biology and cell-free diagnostics [9,30].

Fluorescent protein–based biosensors represent yet another lineage, centered in cell biology and biophysics, where emphasis falls on optical properties, imaging compatibility, and quantitative readouts such as ratiometric signals. FRET-based sensors, in particular, come with their own vocabulary of donor–acceptor pairs, spectral bleed-through, and calibration procedures, and ratiometric response is seen as a major advantage because it normalizes for expression level and imaging conditions [31]. Single-FP sensors, in turn, are valued for simpler constructs and often larger absolute signal changes, but are discussed in terms of limitations in ratiometric quantification and photophysics. Split-protein and chemically induced dimerization systems add a further layer of concepts—such as complementation kinetics, reversibility vs. irreversibility, and background from spontaneous assembly—that are well known in protein interaction and optogenetics communities but only partially overlap with the language used for TF sensors or FRET probes [32].

Each of these modalities thus comes with characteristic hosts (microbial production strains vs. mammalian cells or plants), typical targets (pathway metabolites, neurotransmitters, protein activities), favored performance metrics, and distinct engineering approaches. Because these traditions have grown largely within separate communities, they tend to be optimized, benchmarked, and described using different assumptions and reference points. In practice, this means that a ‘good’ sensor in one modality is not always directly comparable to a ‘good’ sensor in another, and that lessons learned in one lineage are not automatically transferred to others—a modality-level fragmentation that feeds into the broader development landscape discussed below. This is particularly consequential for whole-cell biosensors, where TF-, riboswitch-, and split-protein-based sensors often target chemically related analytes, yet report performance using entirely different metrics—fold induction in one case, switching efficiency or limit of detection in another—making it difficult to judge which scaffold or architecture would be the most productive starting point for a new target.

### 2.3. Project-Specific Development Pipelines: Host, Readout, and Screening Logic

For most biosensor projects, the development pipeline is assembled ad hoc around local choices of host, readout, and screening logic, rather than drawn from a shared, field-wide template, and this contributes directly to fragmentation. Microbial synthetic biology projects often default to *E. coli* or yeast because they are easy to transform and compatible with large libraries, whereas cell biology and neuroscience efforts tend to work directly in mammalian cells or neurons; other communities use plants, fungi, or non-model microbes as their primary context. Expression systems, reporter architectures, and assay formats are then chosen to match local infrastructure—plate readers and bulk fluorescence when only small libraries can be handled, imaging for spatially resolved readouts, droplet microfluidics or growth-based selections where throughput is prioritized—so “standard” pipelines are effectively redefined from lab to lab. Access to equipment, screening capacity, and in-house expertise often shapes these choices as much as any abstract sense of what would be the best general procedure, making it hard for the field to converge on common practices.

Within this broader variability, two contrasting routes to sensor development are especially common, and they are rarely treated as parts of a unified engineering process. In one route, biosensor discovery is driven by bioprospecting: researchers identify natural regulators, receptors, or riboswitches that already respond to a ligand of interest (or a close analogue), transplant them into a chosen host, and refine their performance by tuning expression and regulatory sequences directly in the intended cellular context [33,34]. In the other, more engineering-heavy route, labs start from scaffolds with broad or malleable recognition potential—such as certain allosteric transcription factors, periplasmic binding proteins, monobodies, or receptor–coreceptor pairs—and then use mutagenesis and screening to retune the binding specificity toward new target ligands, often in a development host that is convenient for high-throughput screening rather than matched to the final application [16,35,36]. Both routes can yield effective sensors, but they operate under different assumptions about host context, screening technology, and the extent of sequence-level engineering, and they are documented and optimized in different ways. As a result, even for the same analyte and biosensor modality, the underlying pipelines—from host choice through assay design to sequence refinement—can diverge substantially between groups, reinforcing the sense that biosensor development is a collection of bespoke practices rather than a standardized craft that is shared across the field.

### 2.4. Scaffold-Specific Histories and Idiosyncrasies

Scaffold-specific histories and idiosyncrasies mean that many biosensors come with a ‘local micro-theory’ that lives inside the community that studied the underlying protein or RNA long before it was ever used as a sensor. For example, for soluble abscisic acid (ABA) receptors from plants, more than a decade of structural and biochemical work have mapped the ligand-binding pocket in detail, identified key contact residues, and linked specific side-chain changes to altered specificity, basal activity, or agonist/antagonist behavior [37,38,39,40]. A similar pattern is evident for G protein–coupled receptors, which come with a deep pharmacological and structural literature that defines ligand scope, signaling bias, and trafficking behavior in specific host cells [5,12,41,42,43]. Engineered binding scaffolds such as nanobodies and other single-domain antibodies form yet another class, with detailed structural and affinity data from immunology and structural biology guiding target choice and epitope selection, but with signaling outputs that often require more elaborate designs such as enzyme complementation, quenching, or conformational reporters [44,45]. Comparable scaffold-specific knowledge bases exist for other families, such as periplasmic binding proteins, nuclear hormone receptors, and two-component sensor histidine kinases, each with characteristic constraints on target range, host compatibility, and assay design [36,46,47,48,49,50].

These scaffold histories shape not only which targets a given biosensor can plausibly address but also where and how it can be deployed. Some scaffolds come with broad or “malleable” pockets and are known to accept many chemically related ligands, making them attractive starting points for small-molecule sensing but also raising specificity concerns and requirements for careful off-target testing. Others are inherently narrow or tuned to particular functional groups, limiting the accessible target space but simplifying specificity and toxicity considerations. Certain scaffolds are well tolerated and functional in bacteria, yeast, plants, and mammalian cells, while others are known to misfold, aggregate, or perturb host physiology outside a narrow set of conditions [28]. Even within fluorescent protein–based sensors, different backbones and circularly permuted variants have characteristic brightness, photostability, pH sensitivity, and oligomerization tendencies that strongly influence which imaging setups, expression levels, and fusion partners are viable [32,51].

Because much of this scaffold-specific knowledge is distributed across specialized studies—often focused on native signaling or structural biology rather than biosensing—it tends to be unevenly incorporated into sensor development and reported only partially when a scaffold is repurposed. One group may lean heavily on detailed structural maps of a ligand pocket to design highly targeted mutations, while another uses the same scaffold as a more black-box recognition module, focusing on linker or reporter optimization and inheriting idiosyncrasies they do not fully control. In effect, each widely used scaffold carries its own hidden set of assumptions about chemical scope, host compatibility, and assay design, and these local micro-theories rarely align or interoperate across scaffolds. This scaffold-level idiosyncrasy further fragments biosensor development, because it anchors design practices, target choices, and performance expectations in lineage-specific histories rather than in shared, scaffold-agnostic principles.

### 2.5. Heterogeneous Reporting of Development and Performance

Reporting practices around biosensor development are as heterogeneous as the pipelines themselves, and this heterogeneity spans both how sensors are built and how they are characterized. On the development side, methods sections vary widely in how they describe library design, host strains, induction regimes, and screening conditions, with crucial choices—such as library size and structure, gating strategies, or thresholds used to define ‘responders’—reported at different levels of detail or omitted entirely. On the performance side, studies differ in which metrics they emphasize: fold induction, EC_50_ or apparent Kd, limits of detection, or signal-to-background ratios are each used by different groups as primary descriptors of sensor quality. Which secondary metrics matter beyond these—robustness to cellular and environmental factors or compatibility with specific imaging setups or assay formats—shifts naturally with sensor class and intended application. Differences in units, normalization conventions, and assay conditions compound this further, making direct cross-study comparison difficult even within a single modality.

Efforts to establish reporting norms have emerged in adjacent and overlapping areas. MIFlowCyt, for example, specifies the minimum information required to interpret and reproduce a flow cytometry experiment, directly relevant to the screening and selection side of biosensor development [52]. Norms covering other formats of high-throughput assays, as well as library construction itself, remain sparse. Sensor-class-specific treatments have begun to define what rigorous performance characterization should look like, with relevant sources to draw from existing for transcription factor-based, fluorescent protein-based, and riboswitch-based architectures [29,53,54,55].

Beyond these quantitative details, there is also considerable variation in how new sensors and workflows are named and positioned. New constructs are frequently introduced under bespoke acronyms and branded as standalone tools, sometimes with only modest departures from existing architectures, and with limited effort to map them onto shared categories or to benchmark against representative standards. A sensor developed for a specific metabolite in a particular microbial host, for instance, may be introduced under a unique acronym that encodes the target, the host, or the detection principle—making it difficult for a prospective developer to recognize it as a member of a broader scaffold family, or to locate and compare it with functionally equivalent sensors developed under different names for chemically related analytes. This naming culture reinforces the sense that each biosensor project is qualitatively unique—a one-off invention attached to a particular study—rather than an incremental refinement of common design patterns that can be directly compared, combined, or reused. Taken together, heterogeneous reporting of both development and performance, combined with highly individualized naming and framing, makes it difficult to treat biosensor work as contributions to a shared, cumulative discipline and instead ties many results to the specific labs and contexts in which they were produced.

### 2.6. Limited Shared Repositories and Infrastructure

From the perspective of biosensor discovery, variant libraries are a potential goldmine: each selection campaign implicitly explores a large sequence–function landscape that could, in principle, be re-mined to produce sensors for new target ligands [35,56]. For a given one-off project, however, these libraries are often treated as procedural intermediates on the way to a single optimized sensor, so their long-term storage, documentation and accessibility are often left to informal lab practices. The challenge of preserving and sharing variant libraries and their associated selection histories is not unique to biosensor development—the broader directed evolution and deep mutational scanning communities have similarly grappled with how to make library-level data reusable and comparable across studies, and dedicated infrastructure such as MaveDB has emerged specifically to address this gap [57,58]. In biosensor development, the relevant infrastructure exists in principle—selection campaigns routinely use flow cytometry and deep sequencing platforms that are, in adjacent fields, subject to community reporting norms—but deposition of variant libraries, annotation of selection histories, and sharing of raw sequencing data remain the exception rather than the rule, leaving the cumulative potential of the field’s experimental output largely unrealized—a particularly consequential gap for whole-cell biosensor development, where systematically expanding analyte coverage across chemically related target classes would depend precisely on the kind of cross-campaign scaffold and library reuse that current infrastructure does not support.

This means that a researcher who wants to obtain a genetically encoded sensor for a new target of interest typically has access to finalized constructs but not to the variant libraries and selection histories behind them. Plasmids encoding established biosensors or general-purpose scaffolds are usually available from centralized repositories such as Addgene or DNASU—which specialize in distributing sequence-verified plasmid constructs and curated plasmid collections, usually as plasmid DNA or *E. coli* stocks, together with rich construct-level metadata (vector backbone, selectable markers, full sequence, often growth conditions) [59,60]. Major culture collections such as ATCC and the Leibniz Institute DSMZ likewise provide extensively authenticated microbial strains, but their mission is to act as long-term reference collections rather than as depositories for engineered, pooled variant libraries [61,62]. Domain-specific registries like FPbase aggregate detailed sequence, spectral, and structural information for fluorescent proteins and imaging probes, and offer sophisticated tools for search and optical experiment design, but they do not distribute physical material and typically catalog individual protein states rather than the diversified variant pools used during sensor evolution [63].

There are also important institutional and consortium collections that sit somewhat closer to the library concept. For example, the Yeast ORF Collection and related large-scale ORFeome resources provide genome-wide or near–genome-wide plasmid or strain libraries in yeast, often as *E. coli*-hosted plasmid sets or yeast strains expressing individual ORFs, which can be ordered as curated collections for high-throughput screening [64]. Similarly, DNASU and related repositories distribute full-genome plasmid collections and ORFeome sets as pooled or arrayed libraries, with associated annotation and sequence information [60]. However, these resources are organized around gene content and standardized ORF coverage, not around the specific diversification and selection histories that produced particular biosensor scaffolds. As a result, a researcher seeking to engineer a sensor for a new ligand can find themselves with access to end-point plasmids, reference strains, and rich construct-level metadata, yet without a shared infrastructure for discovering, requesting, and computationally reusing the variant libraries and selection data that could most efficiently guide design.

## 3. Practical Steps Toward Greater Coherence

Taken together, these observations suggest that the main barrier to wider use of genetically encoded biosensors is not a lack of scaffolds or clever designs, but the fact that the process of creating sensors for new target ligands still operates as a largely bespoke craft. In the remainder of this article, we focus on practical, achievable steps that would make biosensor development more efficient, less arbitrary, and easier to adopt beyond specialist laboratories, emphasizing changes that can be implemented incrementally in current workflows rather than speculative future technologies. Where appropriate, we cite published biosensor campaigns that already implement parts of these recommendations as positive examples; these citations are intended to highlight feasible practice, not to be exhaustive or prescriptive.

In particular, we concentrate on biosensor campaigns that reprogram existing scaffolds through mutagenesis and screening [16,56], rather than on studies that develop biosensors via bioprospecting for, and recruitment of, natural scaffolds that respond to the target chemical [10]. We organize these suggestions in roughly ascending order of difficulty: from practices that individual groups can adopt immediately, through shared tools and reporting conventions that facilitate reuse of developmental data and libraries, up to community-level infrastructure such as dedicated data and material repositories.

### 3.1. Adhering to Minimal “Development Record” for Each Campaign

A useful first step toward making biosensor development less bespoke is to ensure that each campaign leaves behind a compact, intelligible “development record,” rather than only a final sensor sequence and a brief Methods paragraph. The goal is not to standardize every experimental detail, but to expose the overall logic of the campaign—what biosensor was developed, how it was obtained, and to what end—so that other groups can quickly understand the path taken and, when useful, build directly on it. Much of this information is already produced in the course of routine work: lab notebooks and protocols capture mutagenesis and assay setup; flow cytometry workspaces encode gating strategies; sequencing runs document the composition of libraries and enriched pools. What is often missing is a lightweight, structured way to surface this information so that it travels with the publication and with deposited materials.

We therefore suggest a single, high-level table—suitable for the main text—as a fast, approach-agnostic way to communicate the essence of a biosensor campaign (Table 1). The table is organized around three simple questions: what was developed, how it was developed, and why—that is, what concrete outcomes and intended applications the campaign delivered. Within this structure, the table captures: the general approach (bioprospecting with architectural refinement versus reprogramming via mutagenesis), the specific scaffold and biosensor class, the diversification strategy, assay and host strain, the selective logic and screening method, and finally the main outcomes and envisaged applications. With a small set of entries, readers can see at a glance whether a given paper, for example, bioprospects a natural transcription factor and refines its genetic context, reprograms an existing FRET sensor by saturation mutagenesis and imaging-based screening, or uses structure-guided design to create a riboswitch followed by growth-based selection. To demonstrate the use of the suggested table format, we provide a worked example based on our previous biosensor development campaign (Supplementary Material, Table S1) [65].

Another practice that can substantially improve transparency and reuse is to document the ontogeny of variant populations—a clear description of how libraries and intermediate pools are derived from one another through successive rounds of selection or sorting. Several recent biosensor studies already implement this, providing in Supplementary Materials descriptive text or tables that allow the reader to track the screening process across stages: which populations were derived from which selection round, what conditions were applied, and what role each pool played in the overall campaign [35,36,56,65,66]. While the high-level table captures the headline “what/how/why” of the campaign, such detailed records in methods or supplementary tables can, where appropriate, document the full population flow for readers who wish to reconstruct or extend the selection trajectory.

### 3.2. Using Shareable, High-Content Readouts for Screening and Selection

Another way to make sensor development more universal and transferable across projects is to converge on a default assay. For mutagenesis-based biosensor engineering, flow cytometry–based screening and sorting are, in many respects, the natural default. They provide single-cell, multiparameter readouts on millions of cells in minutes, which allows developers to both characterize and shape sequence–function landscapes in a way that is very hard to replicate with bulk or growth-based assays [67].

A first advantage is versatility. In a typical campaign, a single FACS run yields fluorescence and scatter data for 10^6^–10^8^ individual cells, each carrying a distinct biosensor variant, under a defined treatment regime. Because the assay volume is small and throughput is high, multiple pre-sorting treatments can be piloted in parallel before each round: for example, aliquots of the same parent library can be exposed to a series of target analyte concentrations, and the minimal dose that elicits a detectable response across the distribution can be chosen as the selective condition to favor sensitivity at low concentrations [65]. Thresholds for sorting are then placed directly on these distributions; by enriching the upper tail of a ligand-treated population, one can target variants with high response amplitude, whereas stringent low-fluorescence gates after growth, in the absence of ligand, enrich variants with low basal activity. Similarly, pre-growth in the presence of off-target ligands followed by selection of low-fluorescence cells makes it possible to actively de-enrich variants that respond undesirably to specific off-targets [56,68,69,70].

A second advantage is that, unlike growth-based selections, sensor activation itself is largely selectively neutral until the moment of sorting. Cells carrying variants with high or low reporter output generally grow similarly over the short timescales of the assay, so false positives and negatives arising from growth defects or compensatory mutations in the reporter system are less common [71]. FACS also handles multiplexing naturally: multiple fluorescence channels allow simultaneous selection on, for example, a sensor channel, a reference or expression marker, and a viability dye, enabling composite gates that directly encode desired transfer function properties (such as high sensor signal at high ligand, low sensor signal at zero ligand, and stable expression levels). Importantly, FACS-based workflows are, by design, screenings rather than one-way selections: “negative” cells remain viable and can be recovered, enabling post hoc characterization or sequencing of both enriched and depleted fractions to map how the library responds to different challenges [72].

A third advantage is that flow cytometry naturally produces standardized, shareable data objects. Each sample generates an FCS (Flow Cytometry Standard) file containing the raw single-cell measurements and acquisition metadata, which can be analyzed by any compliant software and archived for later reanalysis. Community standards such as FCS 3.1 and MIFlowCyt specify how to encode acquisition settings, compensation, and, via associated formats like Gating-ML or ACS containers, the gating strategies used to define selected populations [52]. For biosensor development, this means that developers can, in principle, share not just summary statistics or representative histograms, but the full underlying distributions and the exact gates used for enrichment. Linking these FCS files and gating definitions to the population-level development record (for example, via population IDs in the supplementary table) allows others to re-examine selection regimes, re-fit transfer functions, and mine the same campaigns for different design goals than those pursued in the original study.

Taken together, these features make a strong case for adopting flow cytometry as the default readout in mutagenesis and screening campaigns, wherever it is technically feasible. Doing so would not only improve the efficiency of individual projects, but also help move biosensor development toward a more genuinely disciplinary, data-rich practice rather than a series of isolated crafts.

Flow cytometry, however, will not be the universal solution for every campaign, both because access to instruments and expertise is uneven and because not all biosensor readouts or host systems are naturally FACS-compatible. In such cases, growth-based selections or imaging-based screens can still support disciplined development, provided that treatment conditions, enrichment logic, and population relationships are reported in the same structured way proposed for FACS-based workflows.

For example, growth selections are well suited when large qualitative shifts in biosensor function are needed, imaging screens are preferable when spatial or subcellular context is essential, droplet microfluidics offers high-throughput functional screening for enzymatic or metabolic sensors [73], and paper-based cell-free systems are ideal when working with toxic compounds or when the biosensor is destined for rapid, field-deployable diagnostics outside a traditional laboratory host. Ideally, across all these modalities, the core expectation is identical: make clear how variants were challenged, how ‘winners’ were defined, and how intermediate populations and their data can be located—so that campaigns remain reasonably interpretable and reusable, and so that the scaffolds and assay contexts they validate can serve as starting points for sensors targeting chemically related analytes in future campaigns.

### 3.3. Making Libraries and Related Data Reusable

In biosensor development papers, libraries are often treated as disposable intermediates: they appear as “input material” for a particular selection or screen, and then effectively disappear once a handful of winning variants have been characterized. Yet constructing these libraries—designing the diversity, building and validating the constructs, and running initial selections—represents a substantial investment of time, reagents, and often environmentally costly consumables. From the perspective of future developers, these same libraries are also valuable starting points: a carefully diversified scaffold plus a proven assay context is exactly what one would want when approaching a new target ligand.

To make libraries—along with accompanying strains and related data—easier to reuse, we propose that biosensor papers include, where appropriate, a supplementary table designed to foster reusability–Biosensor Library Reusability (BLR) table. Where the high-level development record introduced above (Table 1) captures the overall logic and outcome of a campaign, the BLR table operates at the level of individual libraries within that campaign, providing the material and contextual detail needed to assess and request a specific resource. This table (Table 2) is structured as a compressed report of each library, letting a prospective developer quickly judge whether it is a plausible starting point for their own ligand of interest. Separate columns can correspond to distinct reusable libraries and their records: the biosensor class, configuration, and modality; the specific scaffold; the ligands that the scaffold is known to recognize naturally and the ligands that have been captured via selection in this library; the compatible assay host and validated assay types; the format in which the physical library is available for sharing (for example, as pooled DNA or in a DH5α-like propagation strain); any accompanying control strains or vectors; pointers to functional and sequencing datasets; a brief history of key selection or curation steps; and embedded stable identifiers for the library, associated strains, plasmids, and data files. In the Supplementary Material, we provide a worked example of a BLR-table based on a previous biosensor development campaign (Table S2) [68].

Consistently using the term “BLR table” in titles and captions will make these tables easy to detect and parse, both by human readers and by search engines or future AI tools. One can imagine a researcher who wants to build a genetically encoded sensor for a particular metabolite starting not from scratch, but by querying “BLR tables from biosensor papers” to find libraries and scaffolds that already recognize related chemotypes, operate in compatible hosts, and are available in a usable physical format. Because the table enforces a small set of common fields—scaffold, native and engineered ligands, diversification strategy and complexity, assay host and type, library format, controls, linked datasets, and selection history—it also provides a ready-made schema for assembling more formal community depositories, the subject of the next subsection.

Framing libraries through BLR tables turns them from transient byproducts into explicit deliverables of biosensor studies. Instead of each paper contributing only a few final sensor variants, it also contributes well-described libraries and assay contexts that can be rediscovered, reanalyzed, and repurposed across projects and laboratories, gradually making the field more interconnected and reducing the need to repeatedly rebuild similar libraries from scratch. Ultimately, making the byproducts of screening efforts more accessible (for example, by using BLR-tables) aligns directly with the self-interest of authors, as subsequent studies re-analyzing or directly building upon these assets will drive long-term citations and lasting impact of their work.

### 3.4. Toward Shared Infrastructure for Biosensor Libraries

A natural next step beyond lab-level practices is to explore what community infrastructure might look like if we took seriously the idea that biosensor libraries and their histories are shared resources rather than per-project consumables. One attractive direction is an “Addgene-inspired” layer for biosensor and synthetic-biology libraries: a library-of-libraries that builds on existing plasmid and strain repositories, but is specialized for pooled, evolvable resources and their associated data [59]. Such an infrastructure could combine familiar elements—stable identifiers, curation, and distribution—with lightweight standards for how biosensor libraries are built, annotated, and accompanied by sequence information and a record of their ontogeny.

In practice, one can imagine a repository track where submitters deposit not only individual constructs, but also curated libraries that conform to basic synthetic-biology conventions (for example, standardized backbones, defined cloning interfaces, and agreed-on reporter architectures), together with a short ontogeny record describing how each library was generated and whether it has undergone any pre-selection. A library might, for instance, be enriched by FACS to deplete auto-fluorescent or constitutively active variants before deposition, so that recipients receive a resource that is immediately “ripe” for screening new target ligands rather than having to re-do generic clean-up steps. Accompanying deep-sequencing data and basic analysis (variant frequencies, enrichment profiles) would allow users to assess coverage and bias, and to select libraries whose apparent chemical or sequence “reach” matches their intended target space.

If such libraries were deposited systematically and linked to their developmental data, they could support more than just incremental reuse. Over time, a library-of-libraries would enable systematic evaluation of which scaffolds and diversification strategies cover which regions of chemical space—including, for whole-cell biosensor development, the ability to ask which existing microbial scaffold libraries already recognize compounds within a target analyte’s chemical class, and where genuine gaps in coverage remain—and would generate precisely the kind of diverse, labeled sequence–function data that machine-learning and computational design approaches require [74,75]. Crucially, from a machine-learning perspective, preserving these complete selection histories captures essential ‘dark data’—including depleted or underperforming variants alongside the ‘winners’—providing the contrastive datasets and full mutational trajectories necessary to benchmark and train predictive generative models [76]. That mapping could be built either through dedicated, large-scale wet-lab efforts, or—perhaps more realistically—by mining deep-sequencing datasets submitted alongside routine campaigns and reanalyzing them in aggregate, in line with FAIR data principles that emphasize findability, accessibility, interoperability, and reusability [77].

There are obvious caveats. Not every library will be suitable for broad redistribution due to biosafety, IP, or host-range constraints; furthermore, archiving complex libraries rather than monoclonal stocks introduces technical risks like clonal drift during long-term storage and subsequent sampling. Any repository would need policies and tooling to handle partial or biased libraries, inconsistent metadata, and evolving standards. Nonetheless, even modest steps in this direction—such as piloting a biosensor-library track within existing repositories, developing minimal annotation templates, and encouraging deposition of both physical libraries and their sequencing data—could substantially increase the long-term value (and potential impact) of individual campaigns without imposing heavy new burdens on developers.

## 4. Conclusions

The central argument of this perspective is straightforward: the main bottleneck in biosensor development is not a shortage of clever scaffolds or creative designs, but the fragmented nature of the development process itself. Biosensor campaigns are conducted in isolation, characterized inconsistently, and reported against a backdrop of local idiosyncrasies that make it difficult to extract transferable insight—let alone transferable material. A reader trying to learn from a published campaign must disentangle scaffold-specific quirks, lab-specific assay conventions, and host-specific tuning choices before any general principle becomes visible; and even then, the biological material that would allow them to build directly on that work is often not archived or shared in a form that is sufficiently accessible. While this central argument remains a conceptual observation within the scope of this review, we propose that a systematic meta-analysis of biosensor development literature could further ground this argument in quantitative evidence.

Some of the factors contributing to the field’s fragmentation are inherent and constructive—the disciplinary diversity that drives biosensor innovation also produces incompatible conventions, benchmarks, and design traditions that no reporting standard can fully reconcile. Nevertheless, the practical steps proposed here—structured development records, flow cytometry as a default assay, reusable library annotations, and community-level deposition infrastructure—are modest in scope and do not require centralized funding or institutional reorganization. Their cumulative effect would be to shift biosensor development from a collection of bespoke crafts toward something more genuinely cumulative: a discipline where each campaign contributes back to a shared substrate of knowledge and material.

A closer look at how biosensor papers are currently structured reveals a tension that makes this cumulative vision harder to achieve than it might appear. Some campaigns are organized primarily around data density, applying deep sequencing across sorted populations to produce sequence-function maps and treating the resulting dataset as the primary deliverable alongside any improved variant [35,72,78]. Others are organized primarily around functional outcome, pushing into new chemical territory and treating the final working sensor as the deliverable, with the selection history compressed into a methods section [16,56,66]. Both modes produce genuine value, but they are strikingly complementary in what they leave behind: the data-rich campaigns generate structured, reusable information over well-characterized chemical ground, while the goal-oriented campaigns extend sensor coverage into genuinely new territory but deposit little of the process that would allow others to build on it.

Shared infrastructure of the kind described here would allow both types of campaigns to contribute to a common resource rather than remaining self-contained. When libraries are systematically annotated, archived, and made available for reuse across groups, they stop being procedural intermediates and become a collectively maintained map of what existing scaffolds can recognize and where the boundaries of that recognition lie. Reuse and reanalysis across campaigns—rather than within them—is what would eventually enable the field to ask which chemotypes are well covered by available scaffolds, which sequence features predict responsiveness to structurally related ligands, and where genuine gaps in chemical space remain—questions that are particularly pressing for whole-cell biosensor development, where expanding analyte coverage systematically across chemically related target classes remains one of the field’s most consequential unmet needs. This kind of cross-campaign analysis is currently out of reach not because the underlying experiments have not been done, but because their outputs are not organized in a way that supports aggregation. Incremental improvements in documentation and deposition would directly unlock it—and in doing so, would provide exactly the kind of diverse, comparable, and context-annotated data that computational design approaches require to generalize beyond the narrow datasets on which they are currently trained.

Progress will be gradual and will depend on changing habits as much as on changing tools. Individual labs can move in this direction by attending, in their reporting, to the needs of prospective follow-up developers—documenting not just what worked, but what was tried, how libraries were structured, and what the selection history looked like. Journals can encourage minimal reporting standards as a condition of publication. Expanding repository infrastructure to accommodate biosensor-specific library tracks would require meaningful effort, but not novel technology—incremental sophistication of existing platforms, with benefits that would build gradually as deposition becomes more routine. Taken together, steps of this kind could help the field consolidate around shared practices without requiring that its deeper disagreements about methods or priorities be resolved first, and over time turn a collection of bespoke campaigns into something more genuinely cumulative.

## Figures and Tables

**Table 1:** High-level development record: a compact, approach-agnostic summary of the overall logic, strategy, and outcomes of a genetically encoded biosensor development campaign. A worked example populating this table for a published reprogramming campaign [65] is provided in Supplementary Table S1.

Category Group	Category	Content
What	General approach	e.g., bioprospecting + refinement of genetic architecture; reprogramming via mutagenesis and selection
	Scaffold (sensing element)	Specific ligand-sensing scaffold (protein, RNA, receptor, etc.), source organism, key known functional features
	Biosensor class/configuration	Class and architecture (e.g., FRET sensor, single-FP intensity sensor, transcription factor, split-TF, riboswitch, protease-based sensor)
How	Diversification approach	e.g., random mutagenesis, rational/targeted mutagenesis, refinement of regulatory sequences, permutation of insertion site, domain swapping)
	Assay and host strain	e.g., *E. coli* FACS screen, yeast growth assay, mammalian cell-imaging, in vitro transcription assay; including main readout and key equipment
	Selective logic and method	e.g., alternating positive and negative FACS, with and without ligand, growth under selection, dual-reporter gating, droplet or plate-based screening
Why	Specific outcomes	e.g., improved dynamic range sensor, sensor retargeted to a new ligand, sequence–activity model that predicts transfer function
	Applications (envisioned or demonstrated)	e.g., metabolic pathway optimization, dynamic feedback control, in vivo imaging, diagnostics, environmental or industrial sensing

**Table 2:** Biosensor Library Reusability (BLR) table: a structured annotation of variant libraries generated during a biosensor campaign, designed to support discovery, assessment, and reuse by prospective developers. A worked example populating this table for a published reprogramming campaign [68] is provided in the Supplementary Material, Table S2.

Category	Content (Example Fields)
Library identifier	Stable short name/ID for the library; version if relevant; pointer to plasmid/library repository entry and plasmid IDs
Biosensor class/configuration/modality	High-level sensor type and architecture (e.g., TF-based transcriptional sensor, FRET sensor, single-FP intensity sensor, riboswitch, split-TF, protease-based sensor)
Scaffold (sensing element)	Specific scaffold name and type (protein, RNA, receptor, etc.), source organism, domain architecture, key functional annotations; stable IDs such as UniProt/GenBank where applicable
Ligand recognized by this and related scaffolds	Ligands recognized by the scaffold in its native context or by related (including engineered) scaffolds; include stable chemical identifiers and citations
Ligands recognized via this library	Ligands successfully recognized after selection with this library (primary target(s), cross-reactive compounds), with brief notes on performance and relevant dataset IDs
Diversification strategy/method	How variation was introduced (e.g., random mutagenesis, targeted/rational mutagenesis, regulatory sequence refinement, permutation of insertion sites, recombination/shuffling); specify theoretical design complexity and, where available, measured library complexity (e.g., CFU, sequencing-based diversity estimates)
Assay host or platform	The biological vehicle or context in which the assay is performed. Host strain(s) or cell line (name, genotype, and relevant genome-inserted modules). For cell-free assays, specify the expression platform and any matrix or material support used (e.g., paper-based substrates, microfluidic chips, lipid vesicles).
Assay type(s) for screening/selection	Validated assay format(s) used to evaluate or partition the library. Specify the experimental modality (e.g., droplet microfluidic sorting, paper-based colorimetric screening, FACS, liquid-handling automated plate readers, or NGS-tracked survival selection), the primary molecular readout (e.g., fluorescence, absorbance, luminescence, cell growth), and the key instrumentation or sorting platforms required
Library availability format	The physical or digital form in which the library is preserved and shared, e.g., pooled plasmid DNA; pooled plasmid in *E. coli* DH5α-like propagation strain; panel of individual clones (with deposit/accession information). For cell-free platforms: stable preparations (e.g., lyophilized DNA on paper substrates, or pre-made extracts).
Control strains/vectors/reference reactions	Defined control materials offered with the library (e.g., empty-vector host, wild-type scaffold strain, non-responsive variant, constitutive reporter strain or plasmid), with strain/plasmid IDs if available. For cell-free platforms: reference materials used to calibrate the assay or determine baseline signals.
Functional data available	Links/identifiers for functional datasets (e.g., dose–response curves, variant fitness scores, sequence–activity maps, summary statistics), including repository accessions
Sequencing data available	Accessions/file IDs for deep-sequencing or other high-content data (e.g., SRA/ENA IDs, internal dataset IDs, sequenced region, read type, minimal analysis notes)
Selection/curation history (compressed)	Short description of key selection/sorting and curation events affecting this library (e.g., elimination of constitutive variants by negative sorting; removal of toxic constructs; major enrichment steps), with pointers to methods sections or figures where details are given
Known scope and limitations	Qualitative notes on where this library works well or poorly (e.g., ligand classes it is biased toward, host dependence, cross-reactivities, stability issues, dynamic range constraints)

## Data Availability

No new data were created or analyzed in this study. Data sharing is not applicable to this article.

## Supplementary Materials

The following supporting information can be downloaded at: https://www.mdpi.com/article/10.3390/bios16060341/s1, Table S1: Worked example of a high-level developmental record table based on a published biosensor development campaign; Table S2: Worked example of a Biosensor Library Reuse (BLR)-table based on a published biosensor development campaign. References are from [79,80,81,82].

## Abbreviations

BLR	Biosensor Library Reuse
CRISPR	Clustered Regularly Interspaced Short Palindromic Repeats
FACS	Fluorescence-Activated Cell Sorting
FP	Fluorescent Protein
FRET	Förster Resonance Energy Transfer
GPCR	G-Protein Coupled Receptor
NGS	Next-Generation Sequencing
ABA	Abscisic acid
PYL	Pyrabacin Resistance-like
Y2H	Yeast Two-Hybrid
