# Supplementary material for: The Fragmented Nature of Biosensor Development: Challenges and Paths to Mitigation
Source: Biosensors (Basel). 2026 Jun 16;16(6):341. doi: 10.3390/bios16060341 (PMC13297061; doi:10.3390/bios16060341)
Supplement: Supplementary file 1 [file biosensors-16-00341-s001.zip › biosensors-4367894-supplementary.pdf]

Supplementary Information

The Fragmented Nature of Biosensor Development: Challenges and Paths to Mitigation

Gil Zimran [1*] and Assaf Mosquna [1,*]

[1] The Robert H. Smith Institute of Plant Sciences and Genetics in Agriculture, the Hebrew University of Jerusalem, 7610000 Rehovot, Israel
* Correspondence: assaf.mosquna@mail.huji.ac.il or gil.zimran@mail.huji.ac.il

Table S1. Worked example of a high-level developmental record table based on a published biosensor development campaign [65].

Category group	Category	Content
What	General approach	reprogramming of ligand specificity via mutagenesis and selection.
	Scaffold (sensing element)	Abscisic acid receptor PYRABACTIN-RESISTANCE-like 3 from Brachypodium distachyon.
	Biosensor class / configuration	Chemically activated Y2H lines. Ligand-induced heterodimer reconstituting a split-transcription factor (GAL4).
How	Diversification approach	Combination of semi-random (targeted saturation mutagenesis of ligand-proximal residues) and random (epPCR) mutagenesis.
	Assay and host strain	FACS using a genome-integrated version of the Y2H-system (strain and vectors)
	Selective logic and method	Toggle-selection, combining high-fluorescence enrichment (following target ligand treatment) to improve sensitivity and response intensity, and low-fluorescence enrichment (following growth w/o ligand) to reduce base-line signal.
Why	Specific outcomes	Mutated BdPYL3 scaffolds responsive to diverse herbicide classes. Low nM detection of alachlor and related herbicides.
	Applications (envisioned or demonstrated)	Detection of alachlor in water or soil samples (10 ppm demonstrated).

Table S2. Worked example of a Biosensor Library Reuse (BLR)-table based on a published biosensor development campaign [68].

Category	Content (example fields)
Library identifier	No stable identifier assigned; Locally managed -80°C storage
Biosensor class / configuration / modality	Chemically activated, genome-integrated Y2H system. Two interactors are each N-terminally fused to different domains of the yeast transcription factor GAL4. Reconstitution of functional GAL4 by ligand induced interaction activates reporter genes.
Scaffold (sensing element)	Abscisic acid receptor PYRABACTIN-RESISTANCE-like 3 from Brachypodium distachyon (locus identifier BRADI1G37810) - fused to GAL4 DNA binding domain;<br>Type-A PP2C phosphatase BdPP2C44 (locus identifier BRADI2G41950) - a ligand-dependent interactor - fused to GAL4 transcription activating domain.<br>Reporter genes are under GAL4-dependent promoters.
Ligand recognized by this and related scaffolds	Abscisic acid and various agonists [79];<br>Multiple structurally diverse herbicides, including alachlor and related chloroacetamides, Bentazon, Fenoxaprop and diuron [65];<br>Mandipropamide and additional pesticides and herbicide-safeners [80];<br>Multiple natural and synthetic cannabinoids and several organophosphates [16];<br>Extensive characterization of ligand-range for a related scaffold identified responsive receptor variants for various plant natural products, drugs, steroids, 2,4,6-trinitrotoluene (TNT), and "forever" per- and polyfluoroalkyl substances (PFAS) [40];<br>Geraniol [81].
Ligands recognized via this library	Alachlor dealkylated metabolite 2-chloro-N-(2,6-diethylphenyl)acetamide [68].
Diversification strategy / method	Targeted saturation mutagenesis of 5 ligand-proximal residues on the background of two single mutations reducing alachlor-responsiveness. Degenerate mutagenic primers were designed to introduce all possible double-mutant combinations.
Assay host or platform	Yeast strain "ST1": Derived from Mav99 [82];<br>GAL1UAS:envyGFP:ADH1term-KanMX4;<br>CT1pro:LexA:ER:haB112:CYC1term–natMX4;<br>PGK1pro:AtATR1:SSA1term–hphMX4;<br>TDH3pro:BdPP2C44:TDH1term–LEU2.

Assay type(s) for screening/selection	Available assay formats:<br>Growth selection by rescue of uracil auxotrophy;<br>LacZ activity;<br>GFP expression (flow-cytometry or plate reader).
Library availability format	Host: E. coli strain DH5a;<br>Vector: p21StBD (addgene #190269) for guided genome-integration in yeast.
Control strains / vectors/ reference reactions	p21StBD-AtPYR1 (no stable identifier assigned; Locally managed -80°C storage) - Strain "ST1" carrying this positive control vector is activated by abscisic acid.
Functional data available	Strain is available on request from the publishing lab, functional data and response curves are published [2,7].
Sequencing data available	No deep sequencing data generated.
Selection / curation history (compressed)	No functional enrichment pre-steps were performed.
Known scope and limitations	The host presents high background fluorescence in plate-reader measurements set to GFP excitation/emission values; PYRACACTIN-RESISTANCE-like receptors are generally biased towards small hydrophobic and semi-hydrophobic molecules.
